# The Impact of Doxycycline as an Adjunctive Therapy on Prostate-Specific Antigen, Quality of Life, and Cognitive Function in Metastatic Prostate Cancer Patients: A Phase II Randomized Controlled Trial

**DOI:** 10.3390/pharmaceutics17040404

**Published:** 2025-03-24

**Authors:** José Guzmán-Esquivel, Hossana S. Garcia-Garcia, Gustavo A. Hernández-Fuentes, Jesús Venegas-Ramírez, Carlos D. Barajas-Mejía, Idalia Garza-Veloz, Margarita L. Martinez-Fierro, Nancy E. Magaña-Vergara, José A. Guzmán-Solórzano, Patricia Calvo-Soto, Oscar N. Avila-Zamora, Mercedes Fuentes-Murguia, Gabriel Ceja-Espíritu, Iván Delgado-Enciso

**Affiliations:** 1 Clinical Epidemiology Research Unit, Mexican Institute of Social Security Institute, Villa de Alvarez, Colima 28984, Mexico; jose.esquivel@imss.gob.mx (J.G.-E.); hosana_19@hotmail.com (H.S.G.-G.); drbarajas.uro@gmail.com (C.D.B.-M.); 2 Department of Molecular Medicine, School of Medicine, University of Colima, Colima 28040, Mexico; gahfuentes@gmail.com (G.A.H.-F.); enyguzsol@gmail.com (J.A.G.-S.); fuentes_murguia@ucol.mx (M.F.-M.); gcejae11@ucol.mx (G.C.-E.); 3 State Cancerology Institute of Colima, Health Services of the Mexican Social Security Institute for Welfare (IMSS-BIENESTAR), Colima 28085, Mexico; onco.avila@gmail.com; 4 Faculty of Chemical Sciences, University of Colima, Coquimatlan 28400, Mexico; nancymv@ucol.mx; 5 Department of Nephrology, Mexican Institute of Social Security (IMSS), General Hospital of Zone No. 1, IMSS, Villa de Alvarez 28984, Mexico; nefrojesusvr@gmail.com; 6 Molecular Medicine Laboratory, Unidad de Medicina Humana y Ciencias de la Salud, Universidad Autónoma de Zacatecas, Zacatecas 98160, Mexico; idaliagv@uaz.edu.mx (I.G.-V.); margaritamf@uaz.edu.mx (M.L.M.-F.); 7 Consejo Nacional de Humanidades, Ciencia y Tecnología (CONAHCYT), Mexico City 03940, Mexico; 8 Coordination of Planning and Institutional Liaison, IMSS OOAD Colima, Colima 28030, Mexico; patricia.calvo@imss.gob.mx; 9 Robert Stempel College of Public Health and Social Work, Florida International University, Miami, FL 33199, USA

**Keywords:** metastatic prostate cancer, doxycycline, prostate-specific antigen (PSA), quality of life, structure–activity relationship (SAR), randomized controlled trial

## Abstract

**Background/Objectives:** Metastatic prostate cancer remains a major clinical challenge, with limited therapeutic options. Doxycycline, a tetracycline antibiotic with anti-inflammatory properties, has shown potential as an adjunctive therapy. This study aimed to evaluate its efficacy in reducing prostate-specific antigen (PSA) levels and improving quality of life in patients receiving standard treatment for metastatic prostate cancer. **Methods:** This phase II, double-blind, randomized controlled trial included 45 participants (aged 57–81 years) assigned to doxycycline (100 mg daily) or a placebo for six months. The primary outcome was the percentage change in PSA levels at 3 and 6 months. Secondary outcomes included quality of life (EQ-5D-5L), cognitive function (Mini-Mental State Examination), and glucose levels. Additionally, a structure–activity relationship (SAR) analysis was performed through an extensive bibliographic review to identify pharmacophores responsible for doxycycline’s biological activity, particularly its tetracyclic core. The SAR analysis included tetracyclines and derivatives, androgen-targeting agents, and other pharmacologically relevant molecules used in prostate cancer therapy. Statistical analysis was conducted using multivariate logistic regression. **Results:** At six months, the doxycycline group showed a median PSA reduction of 60% compared to 10% in the placebo group (*p* = 0.043). A ≥50% reduction in PSA levels was observed in 71.4% of patients receiving doxycycline versus 20.8% in the placebo group (*p* = 0.001), with an adjusted relative risk of 10.309 (95% CI: 2.359–45.055, *p* = 0.002). Quality of life improved, with 7.1% of doxycycline-treated patients reporting poor quality of life compared to 42.9% in the placebo group (*p* = 0.028). A slight improvement in cognitive function was also noted (*p* = 0.037). SAR analysis suggested that the tetracyclic ring of doxycycline may play a crucial role in its observed biological effects. **Conclusions:** Doxycycline demonstrates potential as an adjunctive therapy in metastatic prostate cancer by reducing PSA levels and improving quality of life. The SAR analysis supports the hypothesis that its tetracyclic structure may be responsible for its therapeutic effects. Further large-scale trials are warranted to confirm these findings.

## 1. Introduction

Metastatic prostate cancer (MPC) is one of the leading causes of mortality among men worldwide [1]. In the United States, the incidence rates of MPC increased by 18% over a period of eight years (2008–2016), with an estimated 120,400 men living with MPC in 2018 [2]. This increase is expected to continue, with projections estimating 192,500 men living with MPC by 2030 [3]. Additionally, in 2018, the age-adjusted prevalence of MPC in the U.S. was notably higher in Black men compared to White men (137.1 vs. 62.2 per 100,000 men) [4]. Six years later, studies in Mexico showed that men with prostate cancer tend to present with more aggressive disease at diagnosis, often with metastatic symptoms [5]. Also, studies suggest that Mexican men may have a higher risk of developing prostate cancer, similar to African men, due to genetic and environmental factors [5].

Despite advancements in treatment, the prognosis for metastatic prostate cancer remains poor, highlighting the urgent need for alternative therapies. The standard treatment for this condition includes androgen deprivation therapy (ADT), which aims to reduce testosterone levels, thereby inhibiting the growth of cancer cells. However, as the disease progresses, many patients develop castration resistance, leading to tumor progression and a poor prognosis [6]. Chemotherapy with docetaxel has become an important option for patients with castration-resistant prostate cancer (CRPC), but its side effects limit its effectiveness in some cases [7,8].

An alternative approach in cancer treatment has been the exploration of tetracycline antibiotics, particularly doxycycline, a tetracycline-class antibiotic known for its antimicrobial and anti-inflammatory properties. Doxycycline has demonstrated anticancer properties in preclinical studies across various cancer types, including prostate, breast, duodenal, and colon cancers [9,10]. Its anticancer mechanism is attributed to multiple pathways, including the inhibition of matrix metalloproteinases (MMPs), which are key enzymes in extracellular matrix remodeling and tumor invasion [11,12]. Additionally, doxycycline has been shown to regulate the expression of genes involved in the cell cycle, apoptosis, and cell migration, suggesting that it may interfere with fundamental processes of tumor progression [13].

In recent years, preclinical research has suggested the potential use of doxycycline as an adjunctive treatment for prostate cancer [14]. Specifically, some studies have indicated that doxycycline could enhance the efficacy of standard treatments such as ADT and certain chemotherapeutic agents like cisplatin or docetaxel [14,15]. This approach is based on the premise that doxycycline may reduce tumor inflammation, inhibit metastasis, and improve treatment response. Tetracycline antibiotics, in general, have also demonstrated effects on angiogenesis inhibition, a crucial process in tumor growth, further increasing their potential as additional therapeutic agents [16].

One of the primary biomarkers used to evaluate treatment response in prostate cancer is prostate-specific antigen (PSA). A reduction in serum PSA levels is a common indicator of successful therapeutic response. However, patients who develop castration-resistant prostate cancer (CRPC) experience rising PSA levels despite ADT treatment [17]. Controlling PSA levels is therefore a key objective in the management of metastatic prostate cancer, and various novel therapeutic approaches have been investigated to lower these levels [18]. This includes the repurposing of non-chemotherapeutic drugs with antiproliferative properties, such as common anti-inflammatory agents like mefenamic acid [1].

Preclinical studies have suggested that doxycycline may have a direct effect on prostate cancer cells by reducing their proliferation and migration. For example, in vitro research has demonstrated that doxycycline can induce apoptosis in prostate cancer cells by regulating cellular signaling pathways involved in survival [19,20]. Additionally, some studies have shown that doxycycline can block MMP activity, thereby limiting tumor invasion and metastasis, which are key characteristics of prostate cancer progression [20].

Understanding clinical processes often requires collaboration with other scientific fields. One strategy to obtain rapid insights is in silico modeling, which allows for the estimation of key physicochemical properties, such as solubility, bioavailability, and molecular interactions [21]. Doxycycline is characterized by high lipophilicity, which facilitates its intracellular penetration and interaction with various molecular targets. Some studies have identified significant effects derived from its molecular properties, including its ability to chelate metal ions and modulate mitochondrial function, which could contribute to its anticancer effects [22,23].

Clinical research on the use of doxycycline in cancer remains limited, and it has never been evaluated in prostate cancer patients. However, preliminary findings are promising. The combination of its anti-inflammatory, antiproliferative, and antiangiogenic effects may be key to improving the efficacy of standard therapy while reducing the side effects of conventional treatments. In this context, the primary objective of the present study was to evaluate the efficacy of doxycycline as an adjunctive therapy, compared to a placebo, in patients with metastatic prostate cancer, specifically assessing its impact on biochemical response, measured as a reduction in PSA levels, as well as its impact on patients’ quality of life and cognitive function.

## 2. Materials and Methods

### 2.1. Study Design

This phase II, prospective, double-blinded, two-arm, randomized controlled clinical trial was conducted from August 2020 to March 2024, following the CONSORT guidelines for randomized controlled trials [24]. The study protocol was approved by the Institutional Review Board (IRB) at General Hospital Zone 1 of the Mexican Social Security Institute (IMSS; Colima, Mexico). Written informed consent was obtained from all participants. The clinical trial was registered in the Cuban Public Registry of Clinical Trials (RPCEC) under the identifier DOXCAPROST: RPCEC00000367 (http://rpcec.sld.cu) accessed on 2 August 2020.

### 2.2. Study Subjects

**Figure 1:**
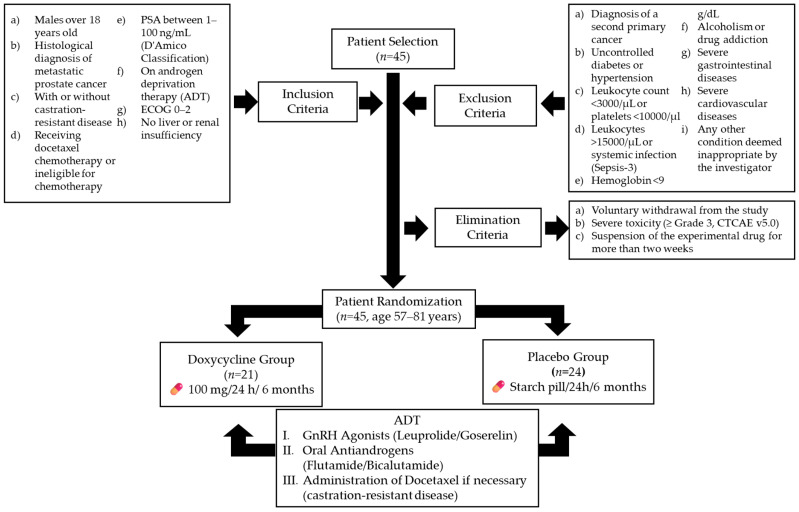
Flowchart of patient selection, randomization, and treatment protocol. ADT (androgen deprivation therapy). Gonadotropin-releasing hormone agonists (GnRH agonists).

The trial enrolled 45 participants from IMSS General Hospital Zone 1 (Figure 1). Inclusion criteria were as follows: male patients over 18 years old with a histological diagnosis of metastatic prostate cancer, with or without castration-resistant disease, based on the Prostate Cancer Clinical Trial Working Group 3 criteria [25]; patients receiving docetaxel chemotherapy or ineligible for chemotherapy; patients with prostate-specific antigen (PSA) levels classified as stages 1–3 in the D’Amico Risk Classification (1–100 ng/mL) [26]; patients undergoing androgen deprivation therapy (ADT) with an Eastern Cooperative Oncology Group (ECOG) performance status of 0–2 [27]; and patients without a history of hepatic impairment (any Child–Pugh classification) [28] or renal dysfunction, with creatinine clearance above 60 mL/min.

Exclusion criteria included the following: diagnosis of a second primary cancer; uncontrolled diabetes or hypertension; leukocyte count below 3000 cells/µL or platelet count below 10,000 cells/µL; leukocyte count above 15,000 cells/µL or signs of systemic infection, based on Sepsis-3 definitions [29]; hemoglobin levels below 9 g/dL; alcoholism or drug addiction; gastrointestinal ulcers; inflammatory bowel disease; ischemic heart disease; chronic heart failure; or any other condition deemed unsuitable by the investigator [30].

Elimination criteria included the following: voluntary withdrawal from the study; severe toxicity (grade ≥ 3) attributed to doxycycline, based on the Common Terminology Criteria for Adverse Events (CTCAE v5.0) [31]; and discontinuation of the experimental medication for more than two weeks, regardless of the cause [32].

After applying the inclusion, exclusion, and elimination criteria, 45 patients aged 57–81 years were randomized into two intervention arms. The doxycycline group (*n* = 21) received 100 mg of doxycycline every 24 h for six months, while the placebo group (*n* = 24) received an identical starch pill for the same duration.

A dosage of 100 mg/day administered orally was selected based on its ability to achieve maximum plasma concentrations (Cmax) of approximately 1700 ng/mL [33]. Previous studies have demonstrated that concentrations as low as 100–1000 ng/mL can reduce the proliferation and migration of prostate cancer cells in vitro [34]. More recently, this dosage, administered over six months, has been shown to be safe and to exert a favorable effect in patients with limited-stage ocular adnexa MALT lymphoma [35] and metastatic colorectal cancer [36]. It is important to mention that both groups continued their ADT regimen, which included either gonadotropin-releasing hormone agonists (leuprolide or goserelin) or oral antiandrogens (flutamide or bicalutamide). Furthermore, the treating physician, blinded to patient assignments, could administer additional treatments as needed, including docetaxel for castration-resistant disease [37,38].

### 2.3. Outcome Measures and Follow-Up

The primary outcome was a clinically significant change in PSA levels at 3 and 6 months. The percentage change in PSA levels was assessed, and biochemical disease progression (PSA increase of ≥25%) was determined using the Prostate Cancer Clinical Trial Working Group 3 criteria. A biochemical therapeutic response was defined as a ≥50% reduction in PSA levels [39].

Secondary outcomes included changes in quality of life (assessed using the EQ-5D-5L questionnaire) and glucose levels. The EQ-5D-5L questionnaire, validated in Spanish, evaluates five domains scored from 0 to 4, with lower scores indicating better quality of life [40]. Cognitive function was measured using the Mini-Mental State Examination (MMSE), where a change of two or more points over six months indicated “relevant cognitive decline” [39]. Additional laboratory assessments included complete blood count and liver and kidney function tests (serum creatinine, BUN, uric acid, albumin, bilirubin, ALT, AST, GGT, ALP, and LDH), monitored every three months as part of routine follow-up. Adverse events were evaluated through patient anamnesis and abnormal laboratory results.

### 2.4. Blinding

Both the research team conducting efficacy assessments and the statistical analysts were blinded to the treatment assignments, as were the patients.

### 2.5. Sample Size

Sample size calculations were based on an expected biochemical response rate (80% PSA reduction), hypothesized to be 40% in the treatment group. This estimate was derived from a historical study on castration-resistant prostate cancer (CRD-PCa) patients treated with mefenamic acid, a nonsteroidal anti-inflammatory drug (NSAID). The control group was assumed to have no biochemical response. Using ClinCalc online software (http://clincalc.com/stats/samplesize.aspx) version 1, accessed 1 September 2020 [41,42], the required sample size for 80% statistical power (α = 0.05) was determined to be 14 patients per arm. Based on the actual biochemical response rates observed at six months, the final statistical power of the study was calculated as 95.2%.

### 2.6. Structure–Activity Relationship Analysis

The structure–activity relationship (SAR) analysis was conducted through an extensive bibliographic review to identify the pharmacophores responsible for the biological activity of doxycycline and related compounds. This analysis included two main groups: (1) tetracyclines and derivatives—doxycycline, chlortetracycline, demeclocycline, minocycline, oxytetracycline, tigecycline, lymecycline, metacycline, and rolitetracycline—and (2) androgen-targeting agents—bicalutamide, flutamide, finasteride, dutasteride, and cyproterone acetate. Additionally, (3) other pharmacologically relevant molecules—docetaxel, leuprolide, and goserelin—were considered due to their therapeutic roles in prostate cancer management. Molecular illustrations were generated using ChemDraw Bio3D software version 12.0 (USA) to optimize the structural configurations of these compounds. Key physicochemical properties, including logP and pKa, were calculated to assess their relevance to bioactivity and pharmacokinetics [22,43,44,45].

### 2.7. Statistical Analysis

Data are presented as the median (first and third quartiles) or percentages. Normality was assessed using the Kolmogorov–Smirnov test, and variance equality was verified with Levene’s test. As several quantitative variables exhibited a non-normal distribution, intergroup comparisons were performed using non-parametric methods, specifically the Mann–Whitney U test, while intragroup comparisons were conducted using the Wilcoxon signed-rank test. Categorical variables were analyzed using Fisher’s exact test. The impact of doxycycline treatment on changes in prostate-specific antigen (PSA) levels at 3 and 6 months was assessed using receiver operating characteristic (ROC) curve analysis. This method was chosen to evaluate the ability of PSA changes to discriminate between patients who received doxycycline and those who received the placebo. Only patients who were concurrently treated with docetaxel were included in this analysis to account for the potential confounding effects of the chemotherapy. Relative risk (RR), number needed to treat (NNT), and 95% confidence intervals (CIs) were calculated to assess the likelihood of achieving biochemical response (PSA decrease of ≥50%) between the doxycycline and placebo groups. Additionally, a multivariate logistic regression analysis was performed to identify factors associated with a biochemical therapeutic response, providing an adjusted relative risk (AdRR). Statistical analyses were performed using SPSS 20.0 (IBM Corp.), with RR and NNT calculations conducted in MedCalc v17.7.2 (MedCalc Software) [46]. Post hoc power analyses and sample size calculations were performed using ClinCalc online software [41]. A *p*-value of <0.05 was considered statistically significant [47].

## 3. Results

### 3.1. Participants

A total of 45 participants were recruited, with a mean age of 73 years (interquartile range: 70.0–78.5). No significant differences were found between the placebo and doxycycline groups in terms of age, body mass index (BMI), Gleason score, glucose levels, docetaxel use, quality of life, or Mini-Mental State Examination (MMSE) score at baseline (Table 1).

### 3.2. Treatment Effects on Prostate-Specific Antigen (PSA) Levels

A significant reduction in PSA levels was observed in the doxycycline group at 6 months compared to the placebo group (Table 2). Initial PSA levels were similar between both groups (median 18.5 in the placebo group vs. 17.0 in the doxycycline group; *p* = 0.473). At 3 months, the median PSA was 17.0 (interquartile range: 9.2–28.0) in the placebo group and 9.0 (interquartile range: 3.5–18.0) in the doxycycline group, without a statistically significant difference (*p* = 0.059). However, at 6 months, patients in the doxycycline group showed a significant reduction in PSA levels (median 7.0, interquartile range: 1.35–14.2) compared to the placebo group (median 16.0, interquartile range: 7.25–22.0; *p* = 0.043). This finding was confirmed in intragroup comparisons, where both the placebo and experimental groups showed a significant reduction in PSA levels at 3 and 6 months (*p* < 0.001).

### 3.3. Change in PSA Levels

**Figure 2:**
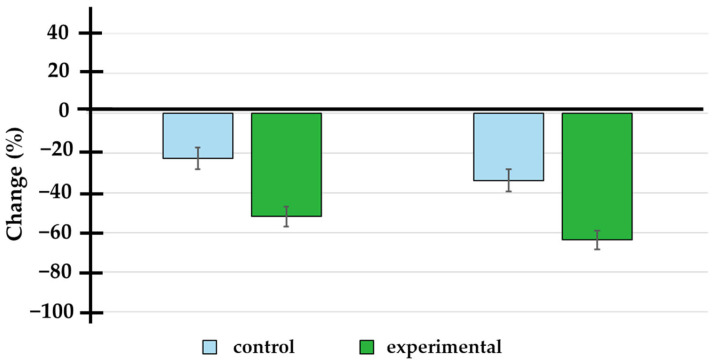
Percentage change in prostate-specific antigen levels at 3 and 6 months of treatment. The doxycycline group showed a significantly higher percentage change (−52.0 ± 22.9% and −63.7 ± 22.1% at 3 and 6 months) compared to the placebo group (−22.6 ± 26.6% and −33.7 ± 27.4% at 3 and 6 months).

A significant reduction in PSA levels was observed in the doxycycline group at both 3 and 6 months compared to the placebo group (see Table 3 and Figure 2). At 3 months, the absolute change in PSA levels was −2.80 (interquartile range: −7.25 to −1.00) in the placebo group, while in the doxycycline group, it was −8.10 (interquartile range: −10.00 to −2.75), with a statistically significant difference (*p* < 0.001). At 6 months, the change was −5.65 (interquartile range: −11.75 to −1.34) in the placebo group and −12.00 (interquartile range: −14.00 to −4.85) in the doxycycline group (*p* < 0.001) (see Table 3). The impact of doxycycline treatment on changes in prostate-specific antigen (PSA) levels at 3 and 6 months was evaluated using ROC curve analysis. To minimize potential confounding factors and avoid biases, only patients who received concomitant docetaxel treatment were included in this analysis. The area under the curve (AUC) was calculated to assess the model’s discriminatory ability. At 3 months, the AUC was 0.200 (95% CI: 0.050 to 0.350, *p* = 0.003), and at 6 months, the AUC was 0.245 (95% CI: 0.081 to 0.408, *p* = 0.012). Although the AUC values were relatively modest, they indicate that the observed PSA changes were statistically significant, further supporting doxycycline’s effect on reducing PSA levels over time. These findings suggest that doxycycline, when administered in combination with docetaxel, may enhance the therapeutic effect on PSA reduction, emphasizing its potential as an adjunctive treatment option.

### 3.4. Therapeutic Response (≥50% Reduction in PSA Levels)

At 3 months, 12.5% of patients in the placebo group achieved a ≥50% reduction in PSA levels, compared to 33.3% in the doxycycline group, although this difference was not statistically significant (RR = 3.500, 95% CI: 0.772–15.878, *p* = 0.151) (Table 4). However, at 6 months, 71.4% of patients in the doxycycline group showed a ≥50% reduction in PSA levels, compared to only 20.8% in the placebo group (RR = 9.500, 95% CI: 2.423–37.248, *p* = 0.001). The number needed to treat (NNT) was 2.0 (95% CI: 1.3 to 3.9) (Table 4). The statistical power of the result, based on the number of patients showing biochemical responses at six months, was calculated to be 95.2%.

### 3.5. Multivariate Logistic Regression Analysis

A multivariate logistic regression analysis was performed to identify factors associated with a biochemical therapeutic response (≥50% reduction in PSA) at 6 months. Treatment with doxycycline showed a significant adjusted odds ratio (AdRR) of 10.309 (95% CI: 2.359–45.055, *p* = 0.002) for achieving a therapeutic response, indicating that patients who received doxycycline had a 10 times higher likelihood of achieving a ≥50% reduction in PSA levels compared to the placebo group (see Table 5). As observed in the context of a multivariate analysis, the administration of docetaxel was not a factor associated with a therapeutic response at 6 months (AdRR 0.79, 95% CI: 0.12–5.00, *p* = 0.808).

### 3.6. Quality of Life and Cognitive Decline

Approximately 46.2% of the patients had poor quality of life at the start of the study, with no significant differences between the placebo and doxycycline groups (*p* = 0.999). However, at 3 months, only 11.1% of patients in the doxycycline group reported poor quality of life, compared to 47.6% in the placebo group (*p* = 0.018). At 6 months, 7.1% of patients in the doxycycline group reported poor quality of life, compared to 42.9% in the placebo group (*p* = 0.028). These results suggest a significant improvement in the quality of life of patients treated with doxycycline compared to the placebo group (Table 6).

In terms of cognitive decline, the doxycycline group showed an improvement in cognitive function in 6 months, with a median improvement of 1 (Q1–Q3: 0–1), while the placebo group showed no change (change of 0, Q1–Q3: 0–1). This difference was statistically significant (*p* = 0.037), suggesting that treatment with doxycycline may be associated with a very slight improvement in cognitive function. There were no adverse events or unexpected alterations in serum analyses or routine blood tests due to doxycycline use. No serious adverse effects were detected from other causes that would require patient exclusion.

### 3.7. In Silico Analysis

While structure–activity relationship (SAR) and physicochemical property analyses are typically conducted during the early stages of drug discovery, we have included them in this study to provide a broader context regarding the potential biological activity of the compounds. Specifically, we aim to highlight key physicochemical characteristics that may influence bioavailability, target interaction, and overall therapeutic potential. Additionally, understanding the SAR allows for insights into possible structural features that could contribute to a new point of view on the biological effects observed.

An analysis was conducted on various physicochemical and pharmacological factors for 17 different molecules, which included antibiotics (mainly tetracycline derivatives) and hormonal agonists/antiandrogens. The analysis examined a broad range of factors, such as molecular weight, number of heavy atoms, aromatic heavy atoms, fraction of sp^3^ carbon atoms, rotatable bonds, hydrogen bond acceptors and donors, logP values (a measure of lipophilicity), and solubility parameters (Table 7).

The physicochemical properties of tetracyclines, androgen receptor antagonists, and hormonal therapies reveal distinct structural and chemical characteristics relevant to their pharmacokinetics and biological activity. Tetracyclines, including doxycycline, chlortetracycline, demeclocycline, minocycline, oxytetracycline, tigecycline, lymecycline, metacycline, and rolitetracycline, generally exhibit moderate molecular weights (MW~440–600 Da), a high topological polar surface area (TPSA~160–240 Å^2^), and significant hydrogen bonding potential due to multiple acceptor and donor sites, contributing to their hydrophilic nature and solubility. Their fraction of sp^3^ hybridized carbons (Csp^3^) varies between 0.32 and 0.52, reflecting differences in molecular rigidity, while rotatable bonds range from 1 to 11, influencing flexibility. LogP values indicate diverse lipophilicity, with iLOGP values spanning from 0 to ~2.7, and some logP values being negative, suggesting variable membrane permeability.

In contrast, androgen receptor antagonists such as bicalutamide, flutamide, finasteride, dutasteride, and cyproterone acetate exhibit lower TPSA (~58–116 Å^2^) and moderate MW (~276–528 Da) levels, with increased lipophilicity as reflected by positive logP values (iLOGP ~1.8–3.8). Their hydrogen bonding capacity is lower compared to tetracyclines, correlating with their distinct interaction profiles with biological targets. The fraction of sp^3^ carbons varies significantly, with finasteride and dutasteride displaying higher values (~0.63–0.83), indicative of a more saturated and flexible structure.

Hormonal therapies, including docetaxel, leuprolide, and goserelin, have the highest molecular weights (~800–1250 Da) and TPSA values (~224–397 Å^2^), suggesting limited membrane permeability and the potential necessity of specialized transport mechanisms. These compounds also show extensive molecular flexibility, as evidenced by a high number of rotatable bonds (~14–20), and their logP values are generally negative, indicating reduced lipophilicity.

**Figure 3:**
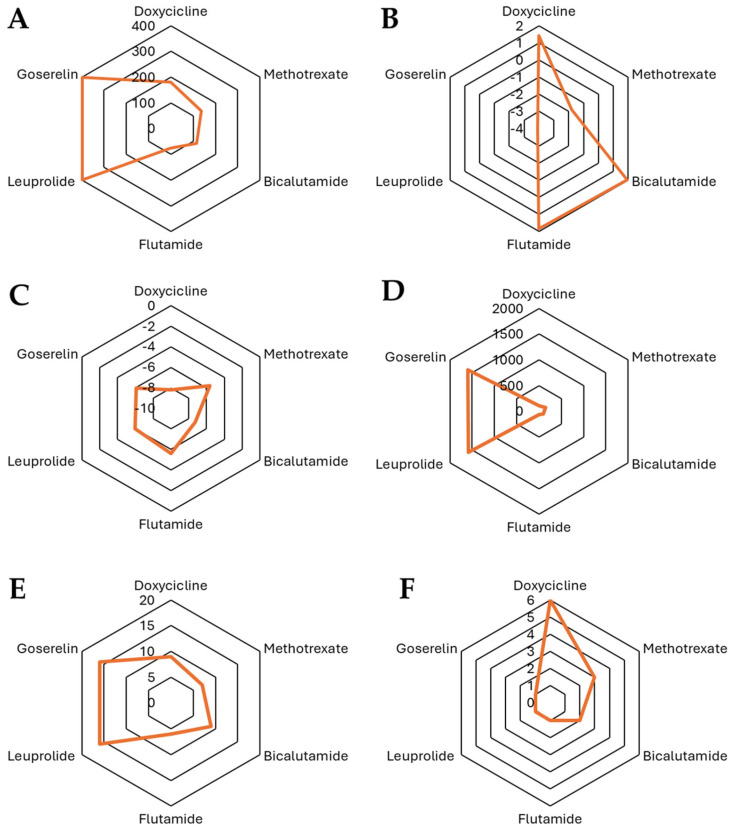
Comparison of the physicochemical properties of the selected compounds: (**A**) TPSA, (**B**) iLOGP, (**C**) log Kp (cm/s), (**D**) MR, (**E**) number of H-Bond acceptors, and (**F**) number of H-bond donors.

In Figure 3, the characteristics are presented from a chemical perspective regarding doxycycline and docetaxel. In this figure, we can observe significant differences in their structure and physicochemical properties, which influence their behavior in biological environments. Doxycycline (MW = 444.43 Da) is an antibiotic from the tetracycline family with a Csp^3^ fraction of 0.41, indicating a combination of planar and saturated regions in its structure. It has only one rotatable bond, suggesting a relatively rigid conformation. Its high number of hydrogen bond acceptors (9) and donors (6), along with a TPSA of 181.62 Å^2^, highlight its highly polar nature and strong hydrogen bonding capacity, which enhances its solubility in aqueous environments. Regarding lipophilicity, its logP values (iLOGP = 1.43, XLOGP3 = 1.19, WLOGP = −0.5, MLOGP = −2.08) indicate a low tendency to cross lipid membranes, suggesting that its absorption may depend on transporters or be preferentially distributed in aqueous compartments. On the other hand, docetaxel (MW = 807.88 Da) is a taxane used in chemotherapy and has a much larger and more complex structure, with a Csp^3^ fraction of 0.56, indicating a higher degree of saturation compared to doxycycline. It has a high number of rotatable bonds (14) with significantly greater conformational flexibility. Unlike doxycycline, docetaxel has 14 hydrogen bond acceptors and 5 donors and a TPSA of 224.45 Å^2^, indicating a higher overall polarity while maintaining a balance between solubility and permeability. In terms of lipophilicity, docetaxel exhibits higher logP values (iLOGP = 3.33, XLOGP3 = 2.81, WLOGP = 2.94, and MLOGP = 1.06), reflecting a greater affinity for lipid phases and suggesting improved passive diffusion across biological membranes compared to doxycycline.

**Figure 4:**
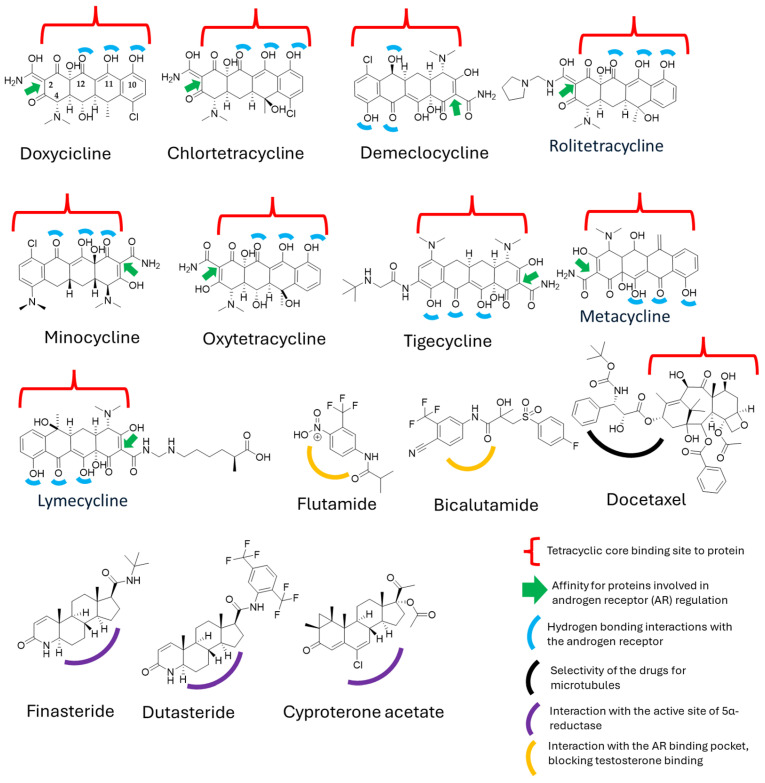
Structural interactions of tetracyclines and derivatives with hormone-binding proteins and enzymes involved in androgen signaling.

When comparing the physicochemical properties of the molecules in question, particularly doxycycline and its derivatives versus docetaxel and the standard treatments for prostate cancer, we observe that they represent opposite extremes in chemical terms, especially regarding polarity and other relevant factors. However, our initial hypothesis about potential physicochemical similarities that could explain the observations in the clinical trial led us to conduct a deeper structure–activity relationship (SAR) analysis. To this end, we generated the structures of both molecules and conducted a literature review, focusing on specific sites where these molecules might exhibit protein affinity or act as key binding sites (Figure 4).

Despite the structural and mechanistic differences between doxycycline and docetaxel, we identified a common feature: the presence of a tetracyclic ring system in both molecules [48,49]. This suggests that, at a structural level, they may share properties that could enable a synergistic effect. In the case of docetaxel, the tetracyclic core is hydrophobic, leading to low water solubility [49], whereas its polar side chains allow for interactions with proteins [49]. On the other hand, doxycycline possesses a polar core due to the presence of functional groups such as hydroxyl (–OH), carbonyl, and amino, which increase its polarity and facilitate its transport in biological environments [50].

Although doxycycline has not been widely explored in this context, some studies have shown that similar ring structures, such as those of anthracene, [51] can form bonds with serum proteins, particularly albumin, by interacting with tryptophan at a binding distance of 15.2 Å, further stabilized by hydrogen bonds [52]. Moreover, studies on anthracene, phenanthrene, and pyrene regarding androgen receptor (hAR) activity have demonstrated a weak antiandrogenic effect, with only slight inhibition of hAR at 10 μM [53]. However, these studies primarily evaluate the structural nucleus, leaving open the possibility that specific chemical modifications could enhance binding affinity, which could be particularly relevant for doxycycline.

Additionally, other structurally similar nuclei have been evaluated for their roles in inflammatory and nociceptive symptoms, such as anthraquinones, which have exhibited significant biological activities [53]. Interestingly, doxycycline has also been found to possess anti-inflammatory and analgesic properties, further supporting the notion that a major part of its biological activity is derived from its tetracyclic core [53,54].

These findings could explain the prolonged distribution of doxycycline in the body, allowing for its gradual release alongside docetaxel while also enhancing its androgen receptor (AR) blocking effects. This suggests that the tetracyclic system may play a crucial role in the interaction between these molecules, although it is not necessarily the sole determinant of their synergistic activity in prostate cancer treatment.

## 4. Discussion

The results of this study suggest that 6-month treatment with doxycycline in patients with metastatic prostate cancer, with or without castration resistance, not only enhances the reduction in prostate-specific antigen (PSA) levels but also has a significant impact on patients’ quality of life and, potentially, cognitive function. These findings are consistent with previous studies that have demonstrated the beneficial effects of antibiotics like doxycycline in various oncological contexts, although the specific use of doxycycline in CRD-PCa has been less explored.

A significant reduction in PSA levels was observed in patients treated with doxycycline compared to the placebo group. At 6 months, 71.4% of patients in the doxycycline group showed a reduction of 50% or more in PSA levels, compared to only 20.8% in the placebo group (RR = 9.500, 95% CI: 2.423–37.248, *p* = 0.001). A multivariate analysis, which included baseline PSA values and the use or non-use of docetaxel, indicated that patients who received doxycycline were 10 times more likely to achieve a therapeutic response compared to the placebo group. Additionally, it was determined that two patients needed to be treated with doxycycline plus usual medical care to achieve one additional patient with a therapeutic response (50% or more reduction in PSA levels) at 6 months (NNT = 2.0, 95% CI: 1.3 to 3.9). These results align with those obtained by previous studies that have suggested that doxycycline may inhibit cell proliferation in various cancer types, including the inhibition of matrix metalloproteinases (MMPs), which play a crucial role in tumor invasion and metastasis [50]. The inhibition of MMPs by doxycycline could explain the decrease in PSA levels, as this marker is related to metastatic activity in prostate cancer [55].

The results support the idea that doxycycline may have a significant biochemical effect, similar to what is observed with other adjuvant treatments, but without severe adverse effects [56]. Doxycycline has a more favorable safety profile, making it an attractive option for patients seeking therapy with fewer adverse risks [57]. Regarding quality of life, the results were notably favorable for patients who received doxycycline, showing significant improvements both at 3 and 6 months compared to the placebo group. At 6 months, only 7.1% of patients in the doxycycline group reported poor quality of life compared to 42.9% in the placebo group (*p* = 0.028). This finding is in line with previous studies that have demonstrated that improvements in the quality of life in cancer patients not only depend on the therapeutic response to the tumor but also the reduction in side effects [10,58].

The underlying mechanism for this improvement may be related to the reduction in tumor burden, which alleviates symptoms associated with advanced prostate cancer. Furthermore, some studies have suggested that antibiotics like doxycycline may have anti-inflammatory properties that could improve symptoms and, consequently, quality of life in cancer patients [58]. Specifically, doxycycline inhibits the activity of matrix metalloproteinases (MMPs), which are involved in inflammatory processes and tumor remodeling, potentially reducing systemic inflammation and thereby improving overall well-being [59].

On the other hand, in terms of cognitive decline, doxycycline treatment showed a small but significant improvement in cognitive levels at 6 months (*p* = 0.037). Although this change was modest, it should be considered that patients with advanced prostate cancer often experience cognitive decline related to factors such as chemotherapy, stress, and side effects from other treatments. The observed improvement in cognitive function in this study may suggest that doxycycline has a neuroprotective effect, possibly through its anti-inflammatory action. Several animal studies have shown that doxycycline can reduce neuroinflammation and improve cognitive function in Alzheimer’s models [60]. Although these findings cannot be directly extrapolated to prostate cancer patients, they suggest a plausible mechanism by which doxycycline could mitigate cognitive decline associated with the disease and its treatments. This result is consistent with findings from other anti-inflammatory drugs, such as mefenamic acid, evaluated in prostate cancer patients [61].

When comparing doxycycline with androgen blockers such as bicalutamide, flutamide, leuprolide, and goserelin from a chemical standpoint, we observe several similarities in their physicochemical properties, which could justify their use in similar therapeutic situations, particularly in the modulation of hormonal activity and its relation to complex biological processes. For instance, both doxycycline and the androgen blockers share common characteristics in terms of solubility and lipophilicity. While doxycycline is more hydrophilic (with a higher TPSA and lower iLOGP compared to some of the androgen blockers), its physicochemical properties are sufficiently close to bicalutamide and flutamide, suggesting that these compounds might engage in similar metabolic and pharmacological pathways [62,63]. These compounds exhibit TPSA and iLOGP values that allow them to interact with similar receptor types and absorption mechanisms in the body. This indicates that, despite belonging to different therapeutic classes, their pharmacokinetic profiles might be compatible [64]

Additionally, doxycycline’s interaction with P-glycoprotein transporters and its ability to cross biological barriers, such as the blood–brain barrier (though with lower permeability compared to some androgen blockers), reinforces the idea that these compounds might exhibit similar effects in terms of bioavailability and tissue distribution [65,66]. Studies have shown that doxycycline interacts with P-glycoprotein, influencing its absorption and distribution [67]. In comparison, bicalutamide and flutamide also exhibit P-glycoprotein interactions that affect their pharmacokinetic properties [68].

At the metabolic level, doxycycline’s interaction with cytochrome P450 enzymes, although to a lesser extent than some of the androgen blockers, could suggest that both groups of compounds compete for or are metabolized via similar liver pathways [69]. Doxycycline has been shown to interact with CYP3A4 and CYP2C19 enzymes [70], while bicalutamide and flutamide also undergo metabolism via these enzymes [71].

However, it is important to emphasize that, although the physicochemical parameters are similar, further studies are needed to confirm whether doxycycline could effectively engage in the same metabolic or physiological pathways as androgen blockers [72]. Additional research should focus on evaluating its potential in the context of hormonal blockers to better understand potential interactions with androgen receptors or mechanisms of hormonal inhibition that could be relevant to its therapeutic use in diseases related to the endocrine or reproductive systems [73]. Further in vivo studies could clarify whether doxycycline could be repurposed or combined with androgen blockers for such indications [74].

Regarding the ability to follow a similar pathway to androgen blockade, some studies have suggested that certain tetracycline derivatives may act as androgen receptor (AR) inhibitors, influencing AR signaling [16,50]. Although tetracyclines are not traditionally classified as antiandrogens, they have been observed to affect AR expression and transcriptional activity, particularly in prostate cancer models [75].

Key structural features associated with AR inhibition include the tetracyclic core (naphthacene–carboxamide), which may interact with steroid-binding proteins and could interfere with AR function by affecting nuclear translocation or transcriptional activity [76]. Some tetracyclines may also modulate AR-related gene expression by inhibiting transcriptional cofactors that stabilize AR-DNA interactions [16]. Among the most studied derivatives, doxycycline and minocycline have been identified as positive allosteric modulators of the PAC1 receptor, inducing plasminogen activators in RT4 cells [77]. These tetracyclines exhibit antibiotic, anti-inflammatory, immunomodulatory, and neuroprotective properties, broadening their potential therapeutic applications [78,79]. Furthermore, the combination of chemotherapy with tigecycline has been proposed as a sensitizing strategy for thyroid cancer treatment [80]. Tigecycline has been shown to inhibit mitochondrial respiration and reduce ATP levels by decreasing mitochondrial membrane potential and inhibiting mitochondrial translation, ultimately leading to oxidative stress and cellular damage [80]. These findings open new perspectives on the therapeutic use of doxycycline and other tetracycline derivatives, particularly in oncology and immunomodulation.

While doxycycline offers promising results in various therapeutic settings, its side effects need to be considered. Some common adverse effects include gastrointestinal discomfort, photosensitivity, and possible esophageal irritation. Additionally, long-term use may lead to the development of antibiotic resistance or alter the microbiota, leading to secondary infections. When compared to other treatments, such as minocycline (which also shows promise as an AR inhibitor), doxycycline tends to have fewer central nervous system (CNS) side effects [81,82]. Minocycline has been associated with more pronounced neurotoxic effects, such as dizziness, headaches, and mood disturbances, whereas doxycycline typically has a more favorable CNS safety profile [83,84]. In cases of cancer resistance, combining doxycycline with androgen blockers or other targeted therapies could offer a more effective strategy. For example, a combination of doxycycline and bicalutamide could target both the androgen receptor pathway and cell proliferation processes, potentially overcoming resistance mechanisms and improving clinical outcomes for patients with prostate cancer. Further studies on combinatory regimens, including doxycycline with traditional androgen blockers, could provide valuable insights into more effective treatment strategies for resistant cancers.

Although the results are promising, the limitations of this study include the relatively small sample size and the limited follow-up duration. Further studies with larger sample sizes and prolonged follow-up are needed to confirm the efficacy and long-term survival of patients. It would also be valuable to investigate the possible molecular mechanisms mediating the effects of doxycycline on tumor progression and cognitive decline. The integration of doxycycline into combination therapies could be an interesting future strategy to enhance the management of patients with advanced prostate cancer.

This study provides promising evidence regarding the efficacy of doxycycline as an adjunctive treatment for patients with metastatic prostate cancer (PCa). Doxycycline showed significant effects in reducing PSA levels, improving quality of life, and presenting a potential cognitive benefit compared to a placebo. The underlying mechanisms of these effects appear to be related to the inhibition of matrix metalloproteinases (MMPs) and the reduction in systemic inflammation, although further research is needed to confirm these mechanisms [50,59].

The findings of this study provide preliminary evidence that doxycycline could be an effective adjunctive treatment in patients with metastatic prostate cancer under androgen deprivation therapy (ADT). The observed improvement in biochemical response, measured by a decrease in PSA levels, suggests that doxycycline may act on tumor cell proliferation by modulating signaling pathways related to tumor growth and invasion [10,14,20]. Previous studies have demonstrated that doxycycline inhibits the activity of matrix metalloproteinases (MMPs), key molecules in extracellular matrix remodeling and metastasis [85,86,87]. This inhibition of MMPs could partially explain the reduction in tumor progression observed in patients treated with doxycycline in the present study.

Additionally, the results related to the improvement in quality of life are significant. The reduction in adverse effects associated with ADT and docetaxel treatment could be related to doxycycline’s anti-inflammatory and neuroprotective properties, which have been reported in other preclinical studies [88]. Chronic inflammation is a key factor in cancer progression and the emergence of treatment-related side effects [89,90], so doxycycline’s anti-inflammatory effects could help improve the overall well-being of patients.

The cognitive function analysis in this study also revealed that patients treated with doxycycline had a significant improvement in their Mini-Mental State Examination (MMSE) scores. This is particularly relevant since patients with metastatic prostate cancer treated with ADT often experience cognitive decline. This finding could be related to doxycycline’s ability to reduce neuroinflammation, a mechanism that has been proposed in previous studies as responsible for its neuroprotective potential [86,91]. This aspect could have important implications for the comprehensive management of cancer patients, as preserving cognitive function improves quality of life and patient autonomy.

However, despite the positive findings, the study has some limitations that should be considered. The sample size is relatively small, which limits the generalization of the results. Furthermore, the follow-up period was only six months, which does not allow for an evaluation of the durability of the observed effects. Larger clinical trials with extended follow-up are needed to confirm these findings, assess the impact of doxycycline on progression-free and overall survival, and explore advanced statistical methodologies, such as survival models or machine learning techniques, to gain deeper insights into treatment response patterns.

## 5. Conclusions

This study provides promising preliminary evidence on the efficacy of doxycycline as an adjunct treatment in metastatic prostate cancer (MPC) patients receiving androgen deprivation therapy (ADT). Doxycycline treatment showed a reduction in PSA levels, improved patients’ quality of life, and resulted in a slight improvement in cognitive function at 6 months. These findings suggest doxycycline’s potential in controlling cancer progression and reducing tumor burden, possibly related to doxycycline’s tetracyclic ring, which shares similarities with other structures and may exert its effects through an antiandrogenic effect. These findings contribute to a broader understanding of metastatic prostate cancer and its treatment, aligning with the existing literature that suggests that doxycycline and similar compounds may reduce inflammation and modulate matrix metalloproteinase activity. This articulation further highlights doxycycline’s potential as a treatment adjunct, offering new insights into therapeutic strategies for metastatic prostate cancer. The confirmation of these results through larger, multicenter trials will be crucial in defining doxycycline’s role in prostate cancer treatment. However, further research is needed to confirm these mechanisms and evaluate long-term safety and efficacy.

## Figures and Tables

**Table 1:** General characteristics and use of traditional and alternative medicine among patients and healthcare professionals at an oncology hospital in western Mexico.

	Usual Medical Care Plus			
Characteristic	All (*n* = 45)	Placebo (*n* = 24)	Doxycycline (*n* = 21)	*p*-Value
Age (years)	73.0 (70.0–78.5)	73.0 (70.0–78.0)	73.0 (69.5–80.0)	0.909 *
BMI	28.3 (26.5–30.7)	28.0 (26.7–30.0)	28.8 (26.7–31.2)	0.542 *
Gleason score	8.1 (7.2–8.5)	8.2 (7.5–8.5)	8.0 (7.0–8.2)	0.852 *
Glucose (mg/dL)	106.0 (91.0–122.0)	100.5 (89.5–120.7)	110.0 (91.7–124.2)	0.391 *
Docetaxel	75.6%	83.3%	66.7%	0.299 **
Poor Quality of Life	46.2%	47.6%	44.4%	0.999 **
MMSE score	22.0 (21.0–25.0)	23.0 (20.2–25.7)	22.0 (21.0–23.5)	0.549 *

Data are presented as the median (first and third quartiles) or percentage. BMI: body mass index; MMSE: Mini-Mental State Examination. * Mann–Whitney U test. ** Fisher’s exact test.

**Table 2:** Evolution of prostate-specific antigen levels at 3 and 6 months of treatment in the placebo and doxycycline groups.

	Control			Doxycycline			
PSA	Median	Q1	Q3	Median	Q1	Q3	*p*-Value *
Baseline	18.50	11.7500	32.00	17.00	7.50	30.00	0.473
3 months	17.00	9.2250	28.00	9.00	3.50	18.00	0.059
6 months	16.0	7.2500	22.00	7.00	1.35	14.20	0.043
P Baseline vs. 3 months **	<0.001			<0.001			
P Baseline vs. 6 months **	<0.001			<0.001			

Data are presented as the median and the first (Q1) and third (Q3) quartiles. * Mann–Whitney U test for intergroup comparison at each time point (control vs. experimental). ** Wilcoxon signed-rank test for intragroup comparison: Baseline vs. 3 months and Baseline vs. 6 months.

**Table 3:** Absolute change from baseline in prostate-specific antigen levels at 3 and 6 months of treatment.

PSA Change	Placebo			Doxycycline			*p*-Value *
	Median	Q1	Q3	Median	Q1	Q3	Control vs. Experimental
3 months	−2.80	−7.25	−1.00	−8.10	−10.00	−2.75	<0.001
6 months	−5.65	−11.75	−1.34	−12.00	−14.00	−4.85	<0.001

Data are presented as the median and the first (Q1) and third (Q3) quartiles. * Mann–Whitney test.

**Table 4:** Percentage of patients with a ≥50% reduction in PSA levels at 3 and 6 months of treatment.

		Therapeutic Response				95% CI		
		*n*	No	Yes	RR	Lower	Upper	*p*-Value
3 months	Placebo	24	87.5%	12.5%	Reference			
	Doxycycline	21	66.7%	33.3%	3.50	0.77	15.87	0.151
6 months	Placebo	24	79.2%	20.8%	Reference			
	Doxycycline	21	28.6%	71.4%	9.50	2.42	37.24	0.001

Therapeutic Response: ≥50% reduction in prostate-specific antigen levels. RR: Relative risk determined using univariate logistic regression.

**Table 5:** Multivariate logistic regression to detect factors associated with biochemical, therapeutic response (≥50% reduction in prostate-specific antigen levels) at 6 months.

		CI95%		
	AdRR	Lower	Upper	*p*-Value
Basal PSA	0.977	0.940	1.014	0.221
Age	0.976	0.870	1.096	0.686
Docetaxel	0.797	0.127	5.002	0.808
Doxycycline	10.309	2.359	45.055	0.002

A multivariate binary logistic regression analysis was conducted to determine adjusted odds ratios (AdORs) with 95% confidence intervals (CIs) and *p*-values. The model included the most clinically relevant variables, such as age, level of basal prostate-specific antigen, docetaxel treatment (which is related to castration-resistant disease), and doxycycline administration.

**Table 6:** Percentage of patients with poor quality of life at baseline and 3 and 6 months of treatment.

Poor Quality of Life	All	Placebo	Doxycycline	*p*-Value *
Baseline	46.20%	47.6%	44.4%	0.999
3 months	30.8%	47.6%	11.1%	0.018
6 months	28.6%	42.9%	7.1%	0.028
P Baseline vs. 3 m **	0.109	0.999	0.014	
P Baseline vs. 6 m **	0.090	0.763	0.014	

* Fisher’s exact test for intergroup comparison. ** Fisher’s exact test for intragroup comparison: Baseline vs. 3 months and Baseline vs. 6 months.

**Table 7:** Physicochemical properties of tetracyclines, androgen receptor antagonists, and hormonal therapies: A comparative analysis.

Molecule	MW	Fraction Csp3	# of Rotatable Bonds	# of H-Bond Acceptors	# of H-Bond Donors	MR	TPSA	iLOGP	XLOGP3	WLOGP	MLOGP
Doxycycline	444.43	0.41	1	9	6	110.91	181.62	1.43	1.19	−0.5	−2.08
Chlortetracycline	478.88	0.41	2	9	6	115.23	181.62	1.92	−0.62	0.33	−1.6
Demeclocycline	464.85	0.38	2	9	6	110.54	181.62	1.55	−0.56	−0.06	−1.82
Minocycline	457.48	0.43	3	8	5	118.57	164.63	1.66	0.05	0.19	−1.6
Oxytetracycline	460.43	0.41	2	10	7	111.95	201.85	0.55	−0.7	−1.51	−2.85
Tigecycline	585.65	0.52	8	10	7	154.95	205.76	2.23	−0.19	0.32	−2.05
Lymecycline	602.63	0.52	11	13	9	151.24	242.98	0	−4.3	−0.56	−4.73
Metacycline	442.42	0.32	2	9	6	110.65	181.62	1.55	−0.24	−0.44	−2.16
Rolitetracycline	527.57	0.52	5	10	6	139.04	170.87	2.69	−0.28	−0.02	−1.4
Bicalutamide	430.37	0.22	7	9	2	93.86	115.64	1.95	2.31	5.34	2.47
Flutamide	276.21	0.36	5	6	1	64.19	74.92	1.85	3.35	4.17	2.03
Finasteride	372.54	0.83	3	2	2	113.18	58.2	3.42	3.03	3.43	3.46
Dutasteride	528.53	0.63	5	8	2	129.94	58.2	3.85	5.37	8.31	5.42
Cyproterone acetate	416.94	0.71	3	4	0	111.96	60.44	3.41	3.64	4.61	3.71
Docetaxel	807.88	0.56	14	14	5	205.25	224.45	3.33	2.81	2.94	1.06
Leuprolide	1237.47	0.24	20	16	1	1590.8	397.28	−3.92	−3.92	−3.83	−3.89
Goserelin	1249.47	0.25	20	16	1	1603.7	397.47	−3.91	−3.91	−3.84	−3.9

Physicochemical properties of tetracyclines, androgen receptor antagonists, and hormonal therapies, including molecular weight (MW), fraction of sp^3^ hybridized carbons (Csp3), number of rotatable bonds, hydrogen bond acceptors (H-bond acceptors), hydrogen bond donors (H-bond donors), molar refractivity (MR), topological polar surface area (TPSA), and logP values (iLOGP, XLOGP3, WLOGP, and MLOGP). Data were analyzed for structure–activity relationships and comparisons between the compound classes. Number (#).

## Data Availability

The original contributions presented in the study are included in the article; further inquiries can be directed to the corresponding author.
